# Enhanced recovery after surgery in Pakistan: a qualitative descriptive analysis of current practices and future directions

**DOI:** 10.1186/s12913-024-11569-w

**Published:** 2024-12-21

**Authors:** Hamza Ahmad, Antonia Arnaert, Waqas Shedio, Omaid Tanoli, Dan Deckelbaum, Tayyab Pasha

**Affiliations:** 1 Center for Global Surgery, McGill University Health Center, 11405 Rue Frigon, Montreal, H3M 2R6 QC Canada; 2 Department of General Surgery, University of Toronto, Toronto, Canada; 3 Department of Cardiac Surgery, Allama Iqbal Medical College, Lahore, Pakistan

**Keywords:** Global surgery, Enhanced recovery after surgery, Qualitative descriptive study, Global health and medicine

## Abstract

Enhanced Recovery After Surgery (ERAS) is a cost-effective perioperative approach that has been shown to shorten patients’ hospital length of stay, improve resource utilization, and reduce postoperative costs for both patients and hospitals. While ERAS has the potential to offer even greater benefits in low- and middle-income countries (LMICs) its successful long-term implementation remains incomplete in Pakistan. This study aimed to explore insights and identify opportunities for implementing ERAS within the local socio-environmental context. A qualitative descriptive approach was employed, using convenience sampling to recruit 11 surgical residents from a public tertiary care hospital in Lahore, Pakistan. Individual semi-structured interviews were conducted. The data collected was then thematically analyzed to capture the residents’ experiences regarding the implementation of ERAS. Acknowledging the benefits of ERAS, participants faced several challenges when implementing ERAS in their respective wards. The participants identified several key opportunities for successful implementation, including enhanced teamwork and collaboration amongst medical teams, improved patient education and compliance towards ERAS, strengthening of peripheral healthcare services, and targeted resource allocation. Even though several challenges identified by the participants were like those highlighted in high-income countries (HICs), unique barriers specific to Pakistan’s healthcare structure and culture also emerged. Further research exploring and highlighting these specific challenges is needed to overcome these core barriers and promote a shift towards a standardized healthcare system focused on improving patient outcomes.

**Supplementary Information:**

The online version contains supplementary material available at 10.1186/s12913-024-11569-w.

## Introduction

Implementation and standardization of ERAS in high-income countries (HICs) has led to a reduced patients’ hospital length of stay and a decrease in the postoperative expenses for both patients and hospitals [1]. Given the growing global burden of non-communicable diseases and the lack of resources in low- and middle-income countries (LMICs) such as Pakistan, the benefits of ERAS are expected to be even greater than those observed in HICs [2]. As such, ERAS has gained interest in Pakistan and several small-scaled randomized control trials (RCTs) demonstrating the benefits of implementing ERAS have been completed. However, despite the local emerging evidence in support of ERAS and the data available from HICs regarding an effective approach to the implementation of ERAS, acceptance and a wide scale integration of ERAS has not been successful in Pakistan [3–5].

Initial experiences of healthcare professionals when implementing ERAS in HICs revealed that a long-term adaptation of ERAS required overcoming several barriers including resistance to change, the support of relevant stakeholders, information provision to patients, availability of allied healthcare professionals and pertinent staff members, aligning different ward cultures, and formation of multidisciplinary teams consisting of various healthcare professionals and management in support of ERAS [6]. Despite these challenges, ERAS implementation in HICs has advanced by using qualitative data to identify key enablers, such as the commitment of appointed champions, cohesive teamwork, stakeholder engagement, supportive leadership, and integration of ERAS into routine practice. Moreover, the appointment of dedicated champions has been crucial for facilitating the transition and ensuring effective long-term implementation [7, 8].

Even though RCTs across Pakistan are demonstrating the benefits of ERAS itself, a paucity of qualitative research aimed at gaining experiences of healthcare professionals’ views regarding ERAS in the specific socio-economic environment of Pakistan could be a rationale for a lack of long-term engagement of ERAS [9]. Therefore, to better understand the potential underlying barriers hindering and opportunities a long-term adaptation of ERAS, this qualitative descriptive study sought out to gain experiences of surgical residents when implementing ERAS at a public tertiary care hospital in Lahore, Pakistan.

## Methods

### Sample and recruitment

The study was commenced in September 2020 and data collection was completed in November 2020. In September 2020, surgical residents and attending surgeons at the tertiary care hospital were introduced to the study, either at grand rounds or using a faculty wide email sent by the head of Cardiac surgery acting as a site coordinator. Participants were advised to privately contact the first and third author to set-up a follow-up call, this was on a voluntary basis. If a participant showed potential interest, the first author elaborated the study purpose and explained the project. The recruitment of the potential participants was done progressively over the month, and by October 2020 all 11 participants had enrolled. Due to the ongoing constraints of Covid-19, lack of attending surgeon availability, and difficulty in accessing administrative staff, a convenience sample of surgical residents was recruited for the study.

### Data collection

Individual interviews were conducted at the hospital during working hours as per participant preference. The interviews lasted between 45 and 60 min, took place in closed private rooms, and were conducted by the first author. To avoid any communication errors, the first author being a native from Pakistan conducted the interviews in Urdu and approached the questions during the interview with non-hierarchical and neutral stance. Each interview was audio-recorded, and at the start of each interview participant signed a consent form and completed a socio-demographic questionnaire. A semi-structured interview guide (appendix 1) with a series of questions to be explored with the participant was used. This interview guide has been provided as a supplementary material. Sample questions included: *What are your current perioperative surgical protocols? Are you aware of enhanced recovery after surgery protocols? Do you use enhanced recovery after surgery protocols in your practice? If you do*,* are they always being implemented? If not*,* why do you think ERAS guidelines are not being adapted?* The interview guide was pilot-tested and validated with the local study coordinator through mock interviews to ensure alignment between the study aim and the interview questions with further refinements being made after the first few interviews [10]. Interviews were conducted until data saturation was achieved.

### Data analysis

The process of data analysis was ongoing and based on an inductive approach [11]. To ensure trustworthiness each transcript was thematically analyzed and supplemented using field notes, journaled by the first author during the time spent in the project setting; documenting feelings, insights, and possible preconceived notions and biases. Transcripts were coded on a line-by-line basis and captions were assigned to recurring concepts. Conceptually similar codes were grouped into meaningful categories and themes. Descriptive statements supported by participant quotes were subsequently formed. To ensure credibility, the first, second, and third authors performed an iterative analysis until a consensus regarding the concepts was achieved. Conducting mock interviews prior to the data collection further enhanced trustworthiness whilst an audit trail to keep track of decisions made during the analysis ensured confirmability.

### Setting

The healthcare system of the tertiary care hospital was much like the rest of Pakistan, adopted from the United Kingdom. Different surgical specialties consisted of several ‘wards’ analogous to surgical units in the North American healthcare system. The number of wards dedicated to a surgical specialty depended on the patient load seeking that respective surgical specialty. Each ward employed several attending surgeons who oversaw the training of a set number of residents. Each surgical ward also had one attending surgeon who was designated as the head of the department (HOD). Overall, standard operating procedures (SOPs) were adapted by the HOD based on the World Health Organization (WHO) and disseminated to the entire staff. Ultimately, the HOD and the attending surgeons oversaw the wards with little input from the administrative body, similar to other public tertiary care centers in Pakistan.

### Ethical considerations

Ethical approval was received from the McGill University’s Research Institutional Board (#A10-B77-20 A) and local administration. Participant informed consent verbal and written was obtained prior to the initiation of the interviews.

## Results

A total of 11 participants enrolled in the study, of which 8 were male and 3 were female. Participants had an average age of 28 years, with a range of 25 to 32 years. Out of the 11 participants, 3 participants were affiliated with Cardiac Surgery, 1 participant was affiliated with Orthopedic surgery, and 6 participants were affiliated with General Surgery. The average years of practice for participants was 4 and the average number of surgeries performed/assisted was 14 in a month. All participants were fluent in Urdu and partially fluent in English. All participants had completed their medical education in Pakistan with 4 having attended workshops on ERAS. Only 1 participant had attended workshops on ERAS outside of Pakistan during an away rotation in the United States of America. Table 1 provides a detailed sociodemographic summary of all the participants.

**Table 1:** Detailed sociodemographic of participants

#	Surgical Specialty	Post-Graduate Training Year/Status	ERAS Knowledge	Attended ERAS workshops	Implement ERAS on wards
1	Cardiac/General	PGY – 4	Yes	No	Partially
2	Cardiac/General	PGY – 1	Yes	Yes	Partially
3	Cardiac/General	PGY – 5	Yes	No	Partially
4	General	PGY – 1	Yes	No	Partially
5	General	PGY – 5	Yes	Yes	Partially
6	General	PGY – 1	Yes	Yes	Partially
7	General	Junior Surgeon	Yes	No	Partially
8	Orthopedic	PGY – 5	Yes	No	Partially
9	General	PGY – 1	Yes	No	Partially
10	Cardiac	PGY – 5	Yes	No	Partially
11	General	Chief Resident	Yes	Yes	Partially

Although ERAS benefits were understood from a patient and hospital perspective, all participants indicated that a complex interplay of cultural, organizational and personal factors impacted implementation of ERAS protocols on the respective wards. Highlighted in the first theme, *A Need for Administrative Oversight*, participants realized that support of ERAS not only by the surgical residents, but also with department leads and hospital leadership would be paramount to successful implementation. This was reinforced in the second theme, *Attending Surgeons’ Role in ERAS Implementation*, where participants recognized and credited certain attending surgeons who were advocating for and making strides towards implementing ERAS. Detailed in the third theme, *Improving Patient Education*, even if certain ERAS guidelines were adapted and encouraged by motivated and interested attending surgeons, more robust patient education programs were necessary for stronger implementation. Moreover, expressed in the fourth theme, *Strengthening Primary Healthcare Services*, participant understood that a gradual shift towards improving primary healthcare services was pertinent to tackle misconceptions, empower and educate patients, and help improve compliance towards ERAS and other protocols without over burdening the tertiary care centers. Finally, emphasized in the fifth theme, *A Resource Pressured System*, participants identified key non-surgical areas including involving allied healthcare workers and an increased availability of tangible resources that could further improve the implementation of ERAS guidelines.

### Need for administrative input

Regardless of the leadership displayed by the attending surgeons, all participants (P1 – P11) highlighted the opportunities for growth within the healthcare culture. Due to the inherent hierarchy involved in healthcare, the attending surgeons led with a strong sense of necessary ownership. According to the participants, having an administrative committee led by senior clinical leadership overlooking the conceptualization and implementation of protocols would provide an opportunity for improved standardization of care between wards. Furthermore, establishing such a committee would not only help with ERAS but also provide a more uniform standardization of care even for non-ERAS protocols amongst the different wards. While attending presence in any healthcare system is variable, developing a team approach and responsible committees would ensure patient safety and efficiency in patient centric models. For participant P10, a continuous oversight and input from the administration would also ensure a long-term implementation of any given protocols; *“With checks and balances*,* adherence to guidelines and protocols will become routine”.* Moreover, according to the participants, involvement of the administration would provide channels for communication between different wards that would ultimately improve interdisciplinary collaboration, which is a crucial aspect for the implementation of ERAS. Participant P3 stated, *“ERAS can be successfully implemented when all teams work together*,* which can be done with increased coordination”.* Participant P6 agreed, *“The challenge can be overcome with coherence between surgeons and other specialists. While the attending surgeon is enthusiastic about ERAS implementation*,* agreement from other specialties is essential*,* because they have their own established practices”.* Hence, standardization of these practices across the various specialties would overall provide a greater coherence and ease for the implementation of ERAS. Participant P8 even expressed the need for standardizing performance audits as a potential solution to further align protocols, *“We conduct quality assessments*,* implement quality improvement committees*,* and have morbidity and mortality rounds. Although guidelines exist for this purpose*,* we lack the necessary meetings and infrastructure to implement them effectively*,* especially if an attending surgeon is expected to do this alone”.*

### Attending surgeons’ role in ERAS implementation

Interestingly, certain attending surgeons were willing to take the lead for the implementation of ERAS guidelines on their respective wards despite a lack of administrative input. For participants P5 and P11, a clear interest was expressed by their attending surgeons towards not only the implementation of ERAS guidelines but also towards teaching the protocols to the residents, which improved adherence to the protocols to a greater extent. According to the participant P5 and P11 this was due to a conscious effort. Participant P5 stated, “*It is a conscious implementation of our professor*. Participant P11 agreed, “*He [professor] tries his best and teaches us as much as possible”.* Not only were the efforts of the professor impacting the short-term implementation of ERAS under his supervision, but it was also instilling a change in the attitudes and practices of the residents, creating a long-term impact. Participant P11 added, *“The idea was instilled by the professor. Now as a senior resident when I see a new resident neglect ERAS*,* I provide them with feedback”.* However, despite the encouragement and extensive knowledge regarding ERAS, participants P5 and P11 expressed facing challenges when implementing certain ERAS guidelines. Participant P11 expressed, *“this should be done [avoid prolonged preoperative fasting]*,* and we know that this is very important for the patient*,* but we are not always able to practice this out of necessity”.*

### Improving patient education

According to the participants (P1 – P11), educating patients on ERAS would greatly support the successful implementation of ERAS, surpassing the effectiveness of any other measure. By providing patients with a better understanding of the post-operative reasons behind specific dietary recommendations such as NPO, clear liquids, and transitioning to a full diet, compliance could be enhanced. Participant P8 expressed, *“Patients sometimes find it confusing to differentiate between liquids and solids. They may raise questions about items like porridge and custard*,* consuming them within the designated 2-hour window*,* which can make things challenging. Instead of simply presenting guidelines*,* we should take the time to explain them clearly.”* Providing patients with pictorial education on the different guidelines was one of the ways the participants were tackling this challenge. Participant P2 mentioned, *“But it is definitely patient education that can help. What we have been trying to do is we provide the patient a pictorial booklet when they are admitted and discharged. We explain regarding their surgery*,* their medications*,* their exercise*,* their diet and even their shower and habits”.* Adding information regarding the ERAS guidelines to such pictorial information booklets could further inform patients about the different elements of ERAS guidelines and improve compliance. Overall, not only did the participants reiterate the importance of educating their patients, they also emphasized pertinent and key areas to focus on. Participant P4 emphasized, *“education of the patient is key. It has to be done properly not like the google education where they can learn anything about their symptoms. Patient education in the sense that they have to be taught about what is being asked of them and why is that being asked”.* When discussing another important element of ERAS, which is the recommendation to minimize the use of opioids for post-operative pain management, participants expressed similar practices. Participants (P1 – P11) acknowledged that opioids are frequently used as the primary choice because they tend to make managing patient complaints easier. To enhance patient education, participants P2, P3, P6, P8 and P9 suggested that educational programs should include family members accompanying the patients because culturally attendants accompanying the patients play a critical role in decision making. Participant P9 mentioned, *“Education of the attendant and counselling of the attendants is equally important”.* By focusing on patient education and involving family members, we can strive to create a more supportive and informed healthcare environment, where patients have a clear understanding of the ERAS process and their role in it. Through effective communication and tailored educational initiatives, we can empower patients and their families to actively participate in their own care, leading to improved outcomes and greater satisfaction with the healthcare experience.

### Strengthening primary healthcare services

Besides promoting shared decision making and patient empowerment through improved communication at the hospital, participants (P1 – P11) all agreed that strengthening the healthcare infrastructure outside the hospital was a key to reinforcing basic principles and improving patient compliance and adherence to ERAS. According to participant P2 currently an ample amount of time and resources were spent addressing misconceptions regarding surgery held by the patients. Participant P2 stated, *“We have patients who come from smaller hospital and are referred to us but they have been informed that after the surgery do not eat for the next 3 days. But we tell the patient that you can eat even 1 day after the surgery. The patient becomes confused*,* stay stuck on their previous misconception and it becomes hard to overcome that”.* Participant P2 further explained that this could be improved if guidelines were standardized amongst the hospitals in the larger metropolitans and the peripheral community hospitals, *“Yes*,* it is a big issue*,* and larger hospital follow most of the guidelines including the WHO guidelines but the smaller hospitals do not follow these guidelines and there are no commonalities”.* Therefore, participants took it upon themselves and counsel the patients at various steps of the hospital admission to improve compliance. Participant P6 explained, *“That is why we do so much counselling. We do the counselling at admission and then at preop and then again right before surgery to help increase compliance”.* Improving primary healthcare in the communities would help improve not only access to healthcare but also provide an effective measure to improve patient compliance and tackle misconceptions without having to over burden the tertiary care centers. Participant P6 added, *“I feel like the compliance after the hospital should be tackled. The compliance after the patient is discharged is very low. We have patient come directly to the surgeons and the hospital for everything. Maybe if we make the general practitioners more available to the public for free for our population that cannot afford to come to the hospital every time*,* maybe then we can have the GP involved who can then help improve the compliance after the hospital.”* Participant P7 shared similar concerns adding that working in a standardized system of healthcare connected with the community hospitals will help reduce the burden at the tertiary care centers and provide more time to individual patients, which would further help with compliance overall, *“If we have a strong GP system placed in this country and we focus on patients outside of the hospital as well*,* then we can definitely tackle this within the hospital too and reinforce these ideas in the hospital and in the periphery equally for all patients”.*

### Resource pressured system

Regardless of the culture present, it was understood by the participants (P1 – P11) that certain elements of the ERAS guidelines were not implementable due to the resource limitations. When discussing elements of the ERAS guidelines focused on food, participants P1 explained, *“the hospital does not have the resources to provide everyone with catered food and meal plans. The food itself in the hospital is from a charitable organization working with the government”.* Even though the food provided to the patients was not ideal for recovering from a surgery, it was the only option the patients had. Participant P2 added, *“We will tell them that they [recovering patients] should not consume a high fatty food but a lot of our patients are from backgrounds that include far areas and low-income backgrounds*,* and they simply cannot afford to get tailored healthier options”.* For participant P3, justifying resources for specific elements of ERAS was not a major focus point because of other more important concerns such as sanitation of the operation theatre. Participant P3 remarked, *“drinks for carbohydrate loading are a concern for later. Only now do we have operation theatres that are up to standards. We have been using water that comes from a regular water tank placed on the top of the building that is not very sanitary”.* For participant P3, P7, and P8, even more general and important elements of ERAS such as early mobilization were not easily implementable due to a lack of allied healthcare professionals. Participant P3 explained, “The biggest issue is also that we do not have physiotherapists. It is usually us [surgical residents] that would ask the patient to get up perform physiotherapy”. Participant P7 added, *“If we have a larger staff and a lower patient load*,* and have a well-trained and educated nursing staff*,* then we can reinforce these things and monitor them over time”.* Nurses were considered an integral part of the team when implementing ERAS and a lack of qualified and educated nurses presented with further challenges; *“You see it comes down all the way to the nurses. Even they are part of the ERAS. They must be educated on these as well. You cannot just implement ERAS through surgeons or anesthesiologists. You must implement it through the entire team” (P8).* Given the patient load encountered, physically it was not feasible for the participants to overlook every patient. Participant P7 added, *“All the load comes directly to the teaching hospitals. Our surgical emergency ward has an outflow of 4000 patients in 24-hours*,* which is much larger than anywhere else”.* For participant P11 another important factor inhibiting the implementation of ERAS was the inability to track and visualize the benefits of any implemented protocols due to a lack of record-keeping and computerized systems. Participant P11 stated, *“the reason why we are not good at implementing evidence-based practice right now is because we do not have a good record keeping option. There is no long-term data available for us to say that yes*,* our practice needs to improve”.*

## Discussions

To our knowledge, this qualitative descriptive study is the first to highlight the perspectives of surgical trainees regarding the opportunities to implementation of ERAS guidelines in Pakistan. Like the results of research conducted in HICs, participants identified areas that could improve the likelihood of implementing ERAS such as the application of standardized treatment plans, more robust healthcare teams including multidisciplinary communication and collaboration for patient centered care, improved tracking mechanisms for quality assurance and improvement strategies, stronger patient education programs geared to an inclusive nature for the patient to be a part of their care plan decision-making, improving support from department heads and hospital leadership, and finally, financial support to implement the above [9, 12, 13]. However, the findings of this study also revealed unique aspects of the healthcare milieu of Pakistan. These aspects of the healthcare system were highlighted through three distinct points that warrant further discussion: (1) establishing interest from the executive leadership and administration, (2) stronger patient education programs and robust primary healthcare delivery, and (3) improved integration of allied healthcare professionals, especially nurse practitioners in ERAS.

Several participants in this study emphasized open communication, improved multidisciplinary collaboration, the implementation of quality assurance and improvement projects to foster an environment conducive to the successful implementation and sustainability of ERAS programs. Coordinating these elements requires effective executive leadership, which can help create and lead programs that initially focus on the isolated implementation of ERAS at selective wards, followed by a wider implementation across the entire hospital, essentially facilitating multilevel leadership alignment [14]. Moreover, it is essential to establish champions and generate interest among key stakeholders who can assume leadership positions to conceptualize and implement strategies for the advancement of ERAS [15]. However, according to participants this preliminary interest and buy-in of executive leadership and administration needed further solidification at the local institution. For many participants, this added interest in promoting ERAS would not only benefit the advancement of the ERAS guidelines but instead also provide coordination and coherence between wards on the current established protocols. With improved coherence and coordination, conceptualizing a mechanism to make ERAS possible will ultimately become easier as leadership of different wards will be more aware of the protocols to follow and utilize this enhanced communication to promote other projects. Nonetheless, it is equally important to acknowledge that getting the executive leadership and administration involved in new projects can be difficult to achieve, especially in a local context of limited resources and financial burdens. As the financial burden increases, even attending surgeons prefer treating patients in the private sector, leaving behind the trainees in the public sector unsupervised and the care process dependent upon on the residents and trainees entirely [16]. It is important to acknowledge that these challenges are not unique to Pakistan or other LMICs. Similar difficulties were encountered during the initial implementation of ERAS programs in high-income countries (HICs), with several programs continuing to struggle. However, unlike HICs, LMICs have an underlying healthcare milieu that makes implementing changes, especially ERAS, more challenging.

Nevertheless, as identified by the participants, several motivated attending surgeons were taking the lead in advocating for ERAS and implementing it on their respective wards. However, a lack of patient education and comprehension of medical practices along with a lacking primary healthcare service structure in Pakistan, created difficulties when implementing standardized healthcare protocols such as ERAS. Again, this was not unique to Pakistan, as implementation programs in HICs identified that patients are not aware of the ERAS guidelines and without a formal education on the protocols, patients rarely follow the guidelines [17]. However, in Pakistan an underlying disconnects between the surgeons and the patients, prevalence of misconceptions about surgical procedures, and a lack of standardization of care between institutions made it even more difficult to convince patients to follow the ERAS guidelines. Improved educational programs that aim to empower patients and promote shared decision making were needed according to the participants. Efforts were being made by the surgical residents and the various departments at educating each and every patient using unique methods. Prior research conducted on this topic in Pakistan elucidates that sharing information with the patient helps improve anxiety and creates a strong patient-physician relationship that could overcome misconceptions. Communication that is warm, normalizes patient fears, and integrates patients’ interpersonal and financial considerations can mitigate anxiety and reduce barriers to accessing care in Pakistan’s public healthcare facilities [18]. However, it was not feasible for the participants to provide catered education to each and every patient under an immense work load. Strengthening primary care services in peripheral areas across Pakistan emerged as a potential solution. The absence of healthcare services in these areas meant that patients had to travel to metropolitan tertiary care centers for surgical services. This geographical disparity, coupled with varying healthcare services between metropolitan and peripheral regions, left patients confused and hesitant to trust surgeons at tertiary care centers. This issue reflects broader health policies at the national level, where a communication gap exists between federal, provincial, and district levels during policy formulation and health planning [19]. As a result, public sector healthcare facilities in peripheral areas of Pakistan are underutilized, highlighting the need to strengthen primary healthcare services in these regions. However, addressing this issue will require gradual, long-term changes in healthcare policies. Nonetheless, conducting research to shed light on this issue can serve as an important initial step.

Lastly, it is important to highlight the role allied health care professionals, especially registered nurses play towards enforcing ERAS. Practically not only do registered nurses spend the most time in contact with the patients but they also spend the most time on the floor observing all aspects of healthcare. Studies exploring the impacts of nurses on the implementation of ERAS highlighted a decline in the compliance towards ERAS practices in the absence of a nurse coordinator because the nurse coordinators are involved in several aspects of patient care such as reducing the consequences of surgical stress, soliciting other actors according to the needs of the patient, anticipating the organization of care and discharge of the patient by staying in touch with a network of liberal nurses, detecting alerts that justify readmission, stimulate patient mobility, and promote recovery of patient autonomy [20]. Hence, it is not a surprise that nurses are often viewed as the face of ERAS [21]. To the contrary, participants in our study expressed being in-charge of all the tasks that a registered nurse, a dietician, or a physiotherapist would traditionally perform. This meant that participants were tasked with not only performing their surgical duties but also overseeing these elements of the healthcare provision for all the patients at the surgical wards, which as they expressed was not feasible in the long-term. Having a nurse coordinator and a nursing staff actively involved in the implementation and integration of ERAS can improve the long-term sustainability of ERAS [22]. However, in Pakistan and other LMICs nurses are not actively involved in establishing or enforcing compliance towards ERAS. Instead, issues such as shortages of nurse educators, a lack of nursing-educated based research, and absence of supportive and productive educational and clinical learning environments results in a lack of highly trained nursing staff in Pakistan [23]. Therefore, a focus towards integrating allied healthcare professionals in the implementation of ERAS by providing them with educational programs and recognizing their concerns could be an important step forward.

### Limitations

This study has some limitations. Although, the experiences of surgical trainees highlighted key opportunities to the implementation of ERAS in Pakistan and provided a deeper understanding of the challenges faced by the healthcare system, the study failed to capture the perspectives of attending physicians, the interdisciplinary healthcare team, and the patients. Without understanding the perspective of the entire healthcare team and patients, it would be difficult to conceptualize solutions that help improve the implementation of ERAS and also ensure the necessary compliance to help yield a successful long-term implementation. Furthermore, the study was conducted a larger tertiary care center in Lahore, Pakistan, which might not be representative of centers in other cities and provinces with unique socio-cultural context especially in smaller cities with more pronounced resource limitations and a lack of patient education. Moreover, the use of volunteers in this study may be a source of selection bias towards trainees knowledgeable on ERAS. Future studies should focus on addressing the perspectives of the entire healthcare team and the relevant stakeholders such as the administration of the tertiary care hospital.

## Conclusion

In conclusion, a comprehensive strategy supporting team building, education, administrative support, standardized protocols, improved teamwork, effective communication, and allocation of adequate resources, will strongly facilitate ERAS implementation in Pakistan with the known patient and institutional benefits including improved surgical outcomes, enhanced patient satisfaction, and reduced healthcare costs. Addressing these barriers will require a collaborative effort from not only the surgeons but also from the administrative team, allied healthcare professionals, and the patients as well.

## Electronic supplementary material

Below is the link to the electronic supplementary material.

Supplementary Material 1

## Data Availability

The datasets generated and/or analysed during the current study are not publicly available because the data is in the form of interviews, which includes several participant identifiers that could potentially endanger participant employment at their respective institutions. However, datasets (Interview Transcripts) are available from the corresponding author on reasonable request.
