# Supplementary material for: Enhanced recovery after surgery in Pakistan: a qualitative descriptive analysis of current practices and future directions
Source: BMC Health Serv Res. 2024 Dec 21;24:1634. doi: 10.1186/s12913-024-11569-w (PMC11663352; doi:10.1186/s12913-024-11569-w)
Supplement: Supplementary file 1 — Supplementary Material 1 [file 12913_2024_11569_MOESM1_ESM.docx]

**Appendix 1: Semi structured Questionnaire**

1. What are your current preoperative surgical practices?
   1. Do you offer preadmission counselling? If so, who is responsible?
   2. Does the preadmission counselling include discharge planning for the patient?
   3. Do you advice and recommend pre-rehabilitation as a preoperative procedure?
      1. BMI
      2. Diabetes
      3. Hypertension
   4. Do you subject patients to preoperative fasting?
      1. How long before the surgery are patients allowed to have solid food? Oral foods?
   5. Do your advice or utilize preoperative carbohydrate loading?
   6. Does the preadmission counselling focus on smoking and alcohol cessation?
      1. If so how long before the surgery does you ask the patient to adhere to qthese guidelines?
   7. Do you perform an antibiotic prophylaxis before the surgery?
      1. At admission or at pre-op?
   8. Do you perform a thromboprophylaxis before the surgery?
2. What are your current intra-operative practices?
   1. Do you use short acting analgesic agents?
   2. Do you monitor body temperature?
   3. Do you carryout active patient warming intraoperatively?
   4. Do you utilize drains during surgery?
   5. Do you maintain water and salt balance during surgery?
3. What are your current post-operative practices?
   1. Do you actively help prevent nausea and vomiting using medication?
   2. Do you use nasogastric tubing for feeding or other purposes?
   3. When do you generally remove the catheter? Is the removal an immediate step post-surgery?
   4. When is solid nutrition resumed or encouraged? When is liquid nutrition resumed or encouraged?
   5. Is chewing a gum recommended post-surgery?
   6. When is mobilization encouraged and assisted with? Are patients encouraged to sit up straight in their bed and move around once they are conscious and alert?
   7. Do you utilize non-opioid oral analgesics or NSAIDs for pain management or utilize intravenous administration of opioids?
   8. Do you keep an audit trail of patient outcomes and satisfaction?
4. Are you aware of the ERAS protocol? What do you believe the ERAS guidelines recommend for your practice?
5. If Yes, where have you heard about them and when?
6. When did you start implementing them in your practice?
7. What are your experiences with using ERAS? Have you used ERAS in your practice in Pakistan or abroad?
8. If yes, is this has been a conscious effort of implementing ERAS protocols in your surgical practice?
9. Has it been difficult to implement ERAS at your hospital?
10. What are the key factors that are important when implementing ERAS? Is population health an important aspect?
11. What are the difficulties or hurdles that you have witnessed or overcome when implementing ERAS?
12. What is in your opinion the biggest hurdle to implementing ERAS in Pakistan?
13. Are you aware of other surgeons utilizing ERAS in their practice?
14. Are you aware of other departments of this hospital utilizing ERAS?
15. If you do not know about ERAS, then given the following information, why do you think you have not been able to implement ERAS?
16. Do you think ERAS should be implemented in Pakistan?
17. Do you think ERAS could be beneficial in a pandemic condition at improving patient care?
18. Do you think telemedicine and tele surgery could be integrated into ERAS to initiate preoperative counselling and physical activity?
19. Would you like to implement ERAS in your surgical practice and explore the benefits of implementing such a protocol?
